# Tuning the brakes − Modulatory role of transcranial random noise stimulation on inhibition

**DOI:** 10.1016/j.brs.2024.03.005

**Published:** 2024-03-06

**Authors:** Alekhya Mandali, Flavie Torrecillos, Christoph Wiest, Alek Pogosyan, Shenghong He, Diogo Coutinho Soriano, Huiling Tan, Charlotte Stagg, Hayriye Cagnan

**Affiliations:** 1 MRC Brain Network Dynamics Unit, Nuffield Department of Clinical Neurosciences, University of Oxford, Oxford, United Kingdom, OX3 9DU; 2 Department of Psychology, University of Sheffield, Sheffield; 3 Neuroscience Institute, University of Sheffield; 4 Department of Bioengineering, Imperial College London

**Keywords:** Cognitive control, transcranial random noise stimulation, beta activity, intermittent bursts

Dear Editor

Cognitive control is an executive function that governs our ability to learn, modify and update actions flexibly [1] and remains challenging to restore with invasive and non-invasive brain stimulation. Though still under debate, inhibitory control is argued to fall within cognitive control [2], with the medial prefrontal cortex (mPFC) being one of the critical structures [3]. Transcranial random noise stimulation (TRNS) modulates cortico-excitability, potentially by altering gamma-aminobutyric acid (GABA_A_) concentration [4], which in the sensorimotor cortex, has been shown to play a vital role in modulating beta rhythms [5]. Building on previous work, we hypothesized that TRNS targeting the mPFC would selectively modulate inhibitory control through GABAergic mechanisms reflected as a change in the beta power and intermittent burst characteristics. To test this hypothesis, we delivered TRNS while recording participants’ neural activity (Figure-1A) using electroencephalogram (EEG) as they performed a modified version of the Go/No-Go task [6].

We recruited 16 participants (one participant dropped due to time constraints) from the general population, who were screened for contra-indications of non-invasive brain stimulation. All participants had normal/corrected vision and were right-handed. The Central University Research Ethics Committee of the University of Oxford approved the study (CUREC-R77362/RE003). The study was undertaken in accordance with the Declaration of Helsinki, and informed written consent was obtained from all participants.

The study followed a within-subject single blinded design, during which participants received either active or sham TRNS in a given session. The participants completed a modified Go/No-Go task (Figure-1B) with a conflict component [6]. We recorded participant’s EEG using a TMSi-Porti amplifier (TMS-International, Netherlands), synchronised to the paradigm via Psychtoolbox. TRNS was delivered using a battery-powered stimulator (DC-Stimulator-PLUS, NeuroConn, GmbH, Germany) via rubber electrodes positioned over F_z_ (Active: outer-ring-4.8cm;inner-ring-2.4cm-diameter) and P_z_ (Return:Rectangular-5x7cm^2^) Figure-1A (supplementary information). The participants were aged 25.8±6.04years and had an impulsivity score of 38.5±7.8 [7]. The Bang-blinding-index for active (0.2) and sham (-0.13) sessions indicated a sufficient level of blinding. The participants reported the presence of expected sensations, such as itching and fatigue at moderate levels.

TRNS stimulation improved inhibitory behaviours, observed as an increase in accuracy in the No-Go condition. Using a two-way Friedman’s non-parametric test (Figure-1C) we compared accuracies at baseline (TRNS:0.95±0.04, sham:0.97±0.04) and after-stimulation (TRNS:1, sham:0.99±0.02), which showed significant differences between the distributions (χ2(3)=15.8,p=0.001) and a significant increase in accuracy for TRNS condition alone (p=0.035) following pairwise comparisons across conditions. A non-parametric Spearman’s correlation showed an inverse relationship between the baseline No-Go accuracy and impulsivity scores (ρ=-0.5, p=0.02), i.e., individuals with higher impulsivity scores made more errors in the baseline-TRNS condition. Furthermore, a Spearman’s correlation showed a positive correlation (Figure-1D) between the impulsivity scores and percentage improvement after TRNS (ρ=0.57, p=0.03), i.e., individuals with higher impulsivity scores had better improvement in their accuracy scores after TRNS but not after sham (ρ=-0.43, p=0.1). There was no effect of stimulation on behaviours concerning Go and Conflict conditions.

To further explore neural signatures driving this improvement in No-Go accuracy, we compared the spectral power over the F_z_ corresponding to the No-Go trials (baseline and after-stimulation) after cue-onset. TRNS increased the spectral power in the beta band(*p*=0.022) over F_z_ (cluster highlighted with an outline in Figure-1E) between 0.5 and 1 seconds after cue onset (time=0) compared to baseline. This increase in spectral power was absent in the sham condition. We then extracted intermittent beta-burst average duration at baseline and after-stimulation for both TRNS and sham conditions. There was a main effect of state (baseline vs after-stimulation: (F(1,13)=6.36, p=0.025)) and interaction (F(1,13)=8.91, p=0.011) but not condition (TRNS vs sham: (F(1,13)=0.16, p=0.69)). A paired sample t-test showed a significant increase in burst duration after TRNS (t(13)=-4.5,*p*<0.001) but not sham (t(13)=0.32,*p*=0.75)(Figure-1F).

Here, we show for the first time that the TRNS induced improvement in stopping behaviours is a function of participants’ baseline impulsivity levels. TRNS had a differential effect on inhibitory control, i.e., participants with higher impulsivity improved more after receiving stimulation. This result supports the notion that the impact of stimulation on behaviour could be a function of baseline performance [8], as observed in other stimulation techniques.

Critically, we show for the first time that this improvement in stopping behaviours after TRNS is mediated through an increase in the low-beta band power (up to 20Hz) (Figure-1E) over the mPFC during No-Go trials. This increase in spectral power coincides with the approximate reaction time during Go and Conflict trials(supplementary) (i.e., expecting a movement). We therefore argue that the observed rise in spectral power after TRNS, specifically in this time window when a movement was observed in Go and Conflict trials, maybe a potential counteractive mechanism to improve inhibition during No-Go trials. Beta was one of the two prominent bands that has been observed over the mPFC, with an ascending oscillatory power across Go, Conflict and No-Go trials [6]. While the precise mechanism through which TRNS modulates beta rhythms remains unknown, taking into account the findings from previous work [4], one could argue that GABA_A_ could drive this modulatory effect. It has recently been shown that oscillatory activity exists as ‘bursts’, i.e., short transient cycles of activity in sensorimotor cortex [9, 10]. Here, we observed beta burst profiles over the mPFC: duration of these temporally localized intermittent bursts was increased by TRNS (Figure-1F). Previously, our research group has shown that burst features in the motor cortex could be modulated by the strength of GABAergic inhibition which inversely correlated with beta burst duration [5]. Therefore, we posit that TRNS may increase the overall burst duration and power by modulating the complex excitatory-inhibitory connectivity of the mPFC via interneurons and GABAergic signalling. However, this requires further confirmation, either using Magnetic Resonance Spectroscopy or paired-pulse protocols.

## Supplementary Material

supp file

## Figures and Tables

**Figure 1:**
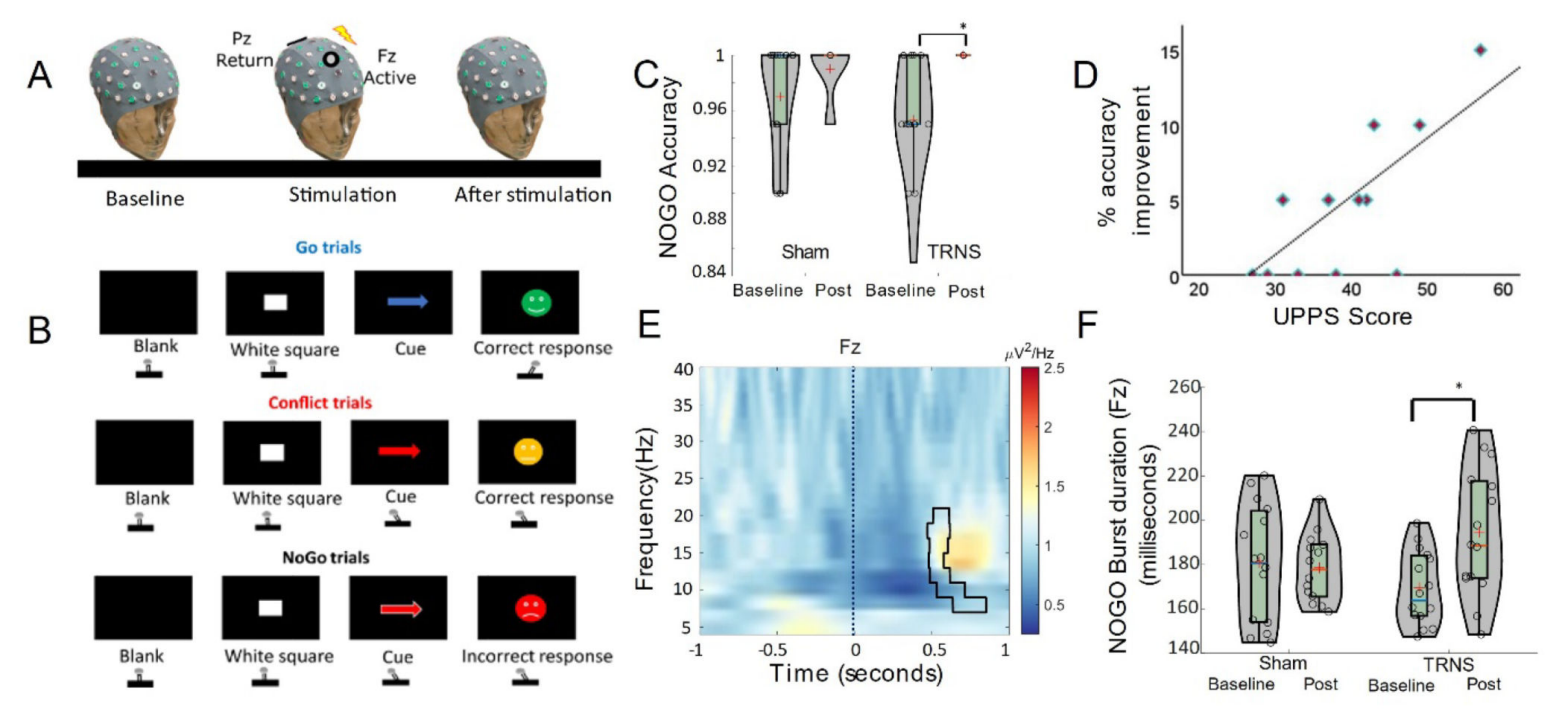
shows a summary of the experimental set up and the cognitive paradigm. (A) shows the sequence of steps in a given session measuring EEGs and behavior at baseline, during and after-stimulation (roughly within 5 minutes of completing the stimulation). TRNS was delivered with the active electrode over F_z_ and the return at P_z_. (B) shows the sequence of events during the cognitive paradigm for Go, Conflict and No-Go trials with feedback for correct, slow-correct and incorrect responses, respectively. (C) shows the No-Go accuracy levels at baseline and after-stimulation for sham and TRNS conditions. (D) shows the improvement in the individual No-Go accuracies as a function of their impulsivity scores after TRNS and (E) shows the F_z_ spectral power after TRNS. The outline shows the increased power in the time-frequency domain when comparing baseline with after TRNS and (F) shows the average burst duration changes across F_z_. The outline in Plot E indicates the significant cluster (p<0.025) and the dotted line indicates the onset of the No-Go cue. * indicates p<0.05

## Data Availability

The analysis code will be made available online.
