# Supplementary material for: Tuning the brakes − Modulatory role of transcranial random noise stimulation on inhibition
Source: Brain Stimul. Author manuscript; Available in PMC 2024 Jun 21. (PMC7616112; doi:10.1016/j.brs.2024.03.005)
Supplement: supp file [file EMS196659-supplement-supp_file.docx]

**Supplementary Methods and Materials**

*Experiment design*

The study followed a within-subject single blinded design, during which participants received either active or sham TRNS. In each session, the sequence of events, as outlined (Figure 1A), were followed. Participants were prepped for the placement of stimulation and recording electrodes. First, participants completed a short version of the cognitive task (up to 6 minutes). TRNS was then delivered while the participants completed the longer version of the cognitive paradigm (up to 20 minutes). The participants either received active (approximately 20 minutes with ramp up/down time of 20 seconds) or sham (turned off after 20 seconds with ramp up/down time of 8 seconds) stimulation. The effect of stimulation was tested using the short version of the task (up to 6 minutes) within 5 minutes of delivering stimulation (TRNS or sham). Participants completed questionnaires on demographics, impulsivity (UPPS measure) [1] and stimulation perception [2]. The stimulation perception questionnaire was completed after each recording session.

*Cognitive Paradigm*

Each participant was seated comfortably in a chair and the task was presented on a 28-inch computer monitor (Dell-Corporation) positioned 50cm at eye-level. Each trial started with the presentation of a blank screen for a variable duration of 1.75 – 2seconds. This was followed by a white square (0.5seconds) which indirectly served as an indicator that subsequently a cue would be presented. The cue could represent Go (arrow in blue), Conflict (arrow in red) or No-Go (arrow in blue or red with white outline), presented in a pseudo-randomized order. Response to the presented cue was measured using a joystick held using their right hand. Participants were expected to respond by moving the joystick in the direction (horizontal movement) of the arrow for Go, the opposite direction for Conflict and not to respond for No-Go trials. Feedback was provided after a response was made, i.e., a green smiley for correct, red for incorrect and yellow for a slow response (>1second) (Figure 1B). This feedback was displayed for 0.5 seconds. Arrow direction was counter-balanced across the 3 cues controlling for direction effects. The task was coded using Psychtoolbox in MATLAB (version MATLAB 2018b, Mathworks, USA) and run on a Dell-Precision-5550 (16GB, Intel i7) Windows-10 system. The proportion of cues was 3:1:1 for Go, Conflict and No-Go, enabling the paradigm to catch uncommon events (Conflict and No-Go). The shorter version of the task had 100 trials (baseline and after stimulation) and the longer version had 300 trials (during stimulation).

*EEG recording*

EEG recordings were limited to 16-channels spanning over the frontal, fronto-central, dominant motor cortex, and mastoid electrodes as they cover the brain regions expected to be engaged in this study [3]. The ground electrode was placed on the left forearm and EEG channels were common average referenced. The EEG recordings and the cognitive paradigm were synchronized using a trigger signal generated by an U3-HV-LabJack (LabJack-Corporation, USA) via the Psychtoolbox. Triggers were sent to the amplifier via Power-1401(Cambridge Electronic Design, UK). Distinctive voltage levels (0-2.5V) were used to mark different events (i.e., blank screen/white square/cue). The trigger signal, and displacement of the joystick were recorded simultaneously with monopolar EEGs using the TMSi-Porti amplifier and were all sampled at 2048 Hz.

*Stimulation*

TRNS was delivered using a battery powered stimulator (DC-Stimulator-PLUS, NeuroConn GmbH, Germany) via conductive rubber electrodes positioned over F_Z_ (Active- ring-4.8cm outer and 2.4cm inner-diameter allowing for EEG measurement) and P_Z_ (Return-Rectangular:5x7 cm^2^) respectively (Figure 1A). The ring electrodes allowed for measurement of neural activity at F_Z_. Each participant’s F_Z_ and P_Z_ positions were estimated using the standard 10-20 system. Before placing the stimulation electrodes, the scalp was scrubbed and prepared to improve conductivity. We applied the Ten20-conductive paste (Weaver and Company, USA) on the stimulation electrodes prior to placing them on the designated positions. Impedance was checked before placing the EEG cap (TMS-International, Netherlands) while paying particular attention to prevent bridging between the stimulation electrodes and EEG electrodes. The mean current (mA) was 1.58±0.44 for TRNS and 0.76 ± 0.3 for sham sessions. The average impedance was under 10kΩ (9 ± 5.15kΩ) across both sessions. The stimulation amplitude was titrated based on each participant’s feedback on tolerance and comfort whilst keeping the amplitude within the allowed safe limits.

*Behavioural analysis*

Behavioral data was analyzed offline using custom made MATLAB scripts (version MATLAB 2018b, Mathworks, USA). Accuracy was determined based on actual and expected joystick movement directions. Reaction time was estimated from the joystick movement: data was epoched such that each segment started 1.5 seconds before and ended 1 second after cue-onset. Reaction time was then estimated as the time point when the joystick position crossed a threshold. The threshold was set at the summation of the mean baseline noise before the cue-onset and 20% of the normalized peak [4]. Baseline noise was included to account for trial-by-trial fluctuations. Trials with response times less than 100ms (i.e., premature, or accidental responses) were excluded from further analysis. Due to high intra-individual variability, all joystick movements were visually inspected and trials with premature movements and direction reversals were excluded. The reaction time per participant for correct and incorrect trials was calculated by averaging across corresponding trials.

*EEG Preprocessing and Analysis*

EEG data was pre-processed offline using custom MATLAB (version:MATLAB-2018b, Mathworks, USA) and EEG LAB(version EEGLAB2021.1-[5]) scripts. Raw EEG data was re-referenced to the mastoid and offset corrected for drifts in the signal. The data was first band pass filtered (high-pass at 0.1 Hz and then low-pass at 100 Hz). Line noise in the signal was removed using an open-source plugin-Zapline (version zaplineplus1.1[6]). Filtered EEG data was subjected to temporal independent component analysis [7] as implemented in EEG LAB [8]. Components representing stereotypical artifacts such as eye blinks and saccades were identified by manual inspection in both time and frequency domains and were removed from the data. On average 2.3 ± 0.2 independent components were removed per participant. Remaining components were then back projected to obtain artifact free channel data. Data was labelled as correct and incorrect per cue type (Go/Conflict/No-Go). Finally, data was visually inspected and segments with muscle artifacts were identified and excluded from further analysis.

Labelled and cleaned data was chunked into epochs using ERPLAB (version:erplab8.3.0 [9] according to the cue type (Go, Conflict and No-Go) and corrected with respect to the average 1 second [-1.5 to -0.5 before cue-onset] inter-trial interval (blank screen). These corrected epochs will be referred to as ‘*ITI-corrected epochs*’ in the subsequent sections. The total length of each epoch was 4 seconds which included 2 seconds before the cue-onset and 2 seconds after. This epoch length was chosen to minimize boundary effects during spectral analysis and was long enough to estimate lower frequency components with sufficient time resolution.

*Time-Frequency analysis*

The ‘*ITI-corrected epochs’* were then processed to calculate spectral power using Fieldtrip [10] in MATLAB. To ensure there were no boundary effects, the spectral power was calculated using a Hanning taper where 6 cycles were chosen per time window for frequencies ranging from 4 to 80 Hz at a resolution of 2 Hz for the time period of -1 to 1.2 seconds (where time = 0 corresponds to cue onset) using the 4 second ITI-corrected epochs. The average power across trials was calculated per participant to identify event related changes (i.e., either event-related synchronization or event-related desynchronization compared to ITI).

*Intermittent Burst analysis*

We studied intermittent properties of the task-evoked oscillations in the beta band using previously validated approaches [11, 12]. First, the peak beta frequency was identified after removing the aperiodic (1/f) component. An opensource package (FOOOF, version:1.0 [13]) was used to identify and remove the aperiodic (1/f) component from the power spectrum. Participants with no distinct oscillatory activity in the beta band were excluded from further analysis. Individual peak frequency was then used to bandpass filter the signal (±2Hz) using a second-order zero-phase lag Butterworth filter. The Hilbert envelope was calculated using the continuous and cleaned EEG data which was then epoched for 4.5 seconds (2seconds before and 2.5seconds after cue-onset) whilst normalizing to the ITI period. All corrected-envelope trials were concatenated to determine the burst threshold (75^th^-Percentile). It should be noted that the threshold was condition specific (here No-Go) similar to the approach used by [14] This threshold was then used to determine the presence of bursts and the effect of TRNS on them. To identify the presence of a burst, the activity was expected to last at least one beta cycle. Estimated burst features (see supplementary: S4) were then averaged to obtain an average feature per participant.

*Event Related potential (ERP) analysis*

The ‘*ITI-corrected-epochs*’ were used for ERP analysis. 20 trials were randomly chosen from the Go condition to ensure comparable signal to noise ratios across all conditions (Go/Conflict/No-Go). The average ERP per participant and condition was calculated and were analyzed for variations in N2 and P3 potentials across mPFC (F_z_) and motor cortex (C_3_).

***Statistics***

*Behavioural analysis*

We ran a 2x2 repeated-measures-ANOVA for state (baseline and after-stimulation) and condition (TRNS and sham) for Go and Conflict trials for both accuracy and reaction times for correct trials. Post-hoc tests were Bonferroni corrected. For error trials, participants with no errors were excluded and mean reaction times were calculated.

Performance in No-Go trials (accuracy) was tested using a non-parametric Friedman’s 2-way analysis as the data did not pass the Kolmogorov-Smirnov normality test. Pairwise comparisons between conditions were Bonferroni corrected to adjust for multiple comparisons.

*Cluster based statistics for spectral data*

Statistical difference between two time-frequency power spectrums was evaluated using a cluster-based Monte Carlo non-parametric method, using the MATLAB based fieldtrip package. Here, a paired t-test on each time-frequency combination (power-spectrum A and power-spectrum B) was compared and clusters were identified and labelled as significant if its summed t-statistic value exceeded 97.5% of the randomized distribution (p < 0.025) to test for both positive and negative differences. Neighboring channels for each of the 15 EEG channels were calculated using a triangulation method [15] for details) resulting in an average of 4.9 neighbors per channel. The procedure was repeated 3000 times while randomly exchanging labels between the 2 power-spectrums for each subject between for the 0-1second time period.

*Comparing non-cue and cue-evoked activity at baseline*

The power spectrums corresponding to the non-cue period (1-second before cue-onset) and cue period (1-second after cue-onset) were first computed and extracted using Fieldtrip. We then collapsed the power spectrum corresponding to Go, Conflict and No-Go events similar to the approach used in [16] and statistically compared them (non-cue vs cue periods) using the cluster based approach.

*Effect of stimulation on Cue-specific activity*

The effect of stimulation on spectral power was estimated by comparing the power spectrums corresponding to baseline condition and after-stimulation between 0 and 1 seconds after cue onset and frequency range 8-20Hz. This frequency range was selected based on the cluster range observed while comparing baseline and cue-evoked power spectrums (Figure S1).

*Cluster based statistics for ERP data*

Statistical difference between two time-series was evaluated using a cluster-based Monte Carlo non-parametric method, using the MATLAB based fieldtrip package. The procedure was repeated 3000 times while randomly exchanging labels between the 2 conditions for each subject between 0 and 0.5 seconds.

*Burst Analysis*

Various burst features such as duration, amplitude, and number of bursts were compared by calculating the median across trials for each participant for F_z_ and C_3_. Extracted values were then compared using a 2x2 ANOVA (baseline and after stimulation) for TRNS and sham conditions. Pairwise comparisons between conditions were Bonferroni corrected to account for multiple comparisons.

**Results**

*Baseline behavior*

We first evaluated behavior at baseline (before receiving stimulation) by calculating the accuracy and reaction time (seconds) during Go and Conflict trials. We compared accuracy and reaction times during Go and Conflict trials at baseline by collapsing across TRNS and sham conditions. As expected, accuracy for the conflict condition (0.94 ± 0.06) was significantly lower (t(29) = 3.72, *p*<0.001) than Go (0.98 ± 0.01) (Figure S1A). Similar to accuracy, we observed a significant difference between Go and Conflict reaction times (t(29) = -16.29, *p*< 0.001): trials involving conflict (0.59 ± 0.06) were significantly slower than Go (0.49 ± 0.04) (Figure S1B).

*Baseline vs Evoked Time-frequency Analysis*

We compared the spectral powers evoked due to presentation of a cue when collapsed across Go, Conflict and No-Go trials for baseline TRNS session compared to non-cue period. In line with the previous literature, we observed an increase (See Supplementary Figure-S2) in spectral power in theta and a decrease in lower-beta bands (p<0.005) over F_Z_ (Figure S1C) and C_3_ (Figure S1D) after the onset of a cue (Go/Conflict/No-Go), compared to the non-cue period.


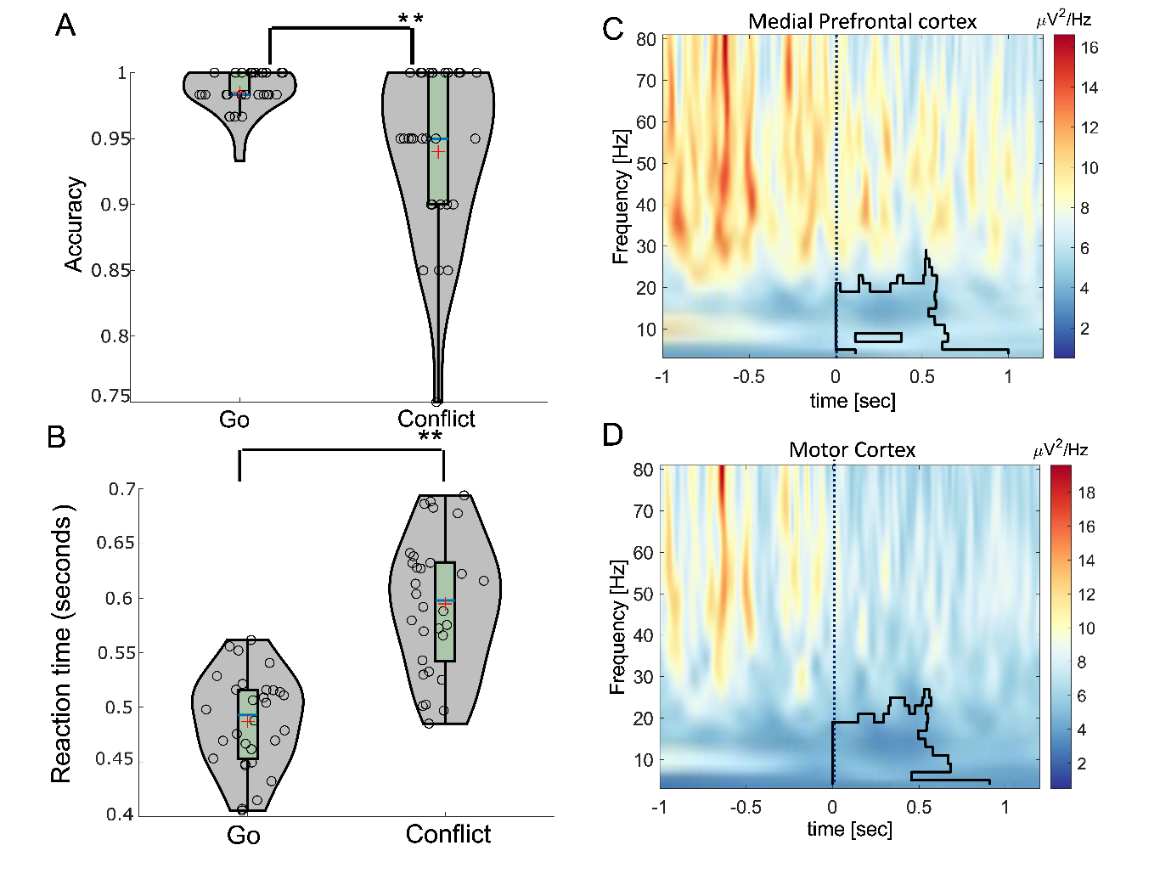


Figure S1 shows the baseline behaviour and evoked activity. (A) and (B) indicate the baseline accuracy and reaction time for correct Go and Conflict trials collapsed across baseline TRNS and sham sessions and (C) and (D) show the spectral powers evoked due to presentation of a cue when collapsed across Go, Conflict and No-Go trials for baseline TRNS session. The outline corresponds to significant clusters computed using the Montecarlo non-parametric test (p< 0.025) when comparing evoked activity between [0 1] seconds with non-cue period of the same window length [-1.0 0] seconds where ‘0’ is cue-onset. The red cross in (A) and (B) indicates the mean of the sample and blue horizontal bar indicates the median. ** indicates p<0.001.

Figure S2 shows the difference in power spectral density between cue (0 to 1 second) and non-cue period (-1 0) in seconds for F_z_ and C_3_.

*Effect of stimulation on behaviour*

*Effect of stimulation on Go/Conflict behaviour*

We next explored the effect of stimulation during Go and Conflict trials on accuracy and reaction times (2x2 repeated-measures-ANOVA–state: baseline and after stimulation and condition: TRNS and sham).


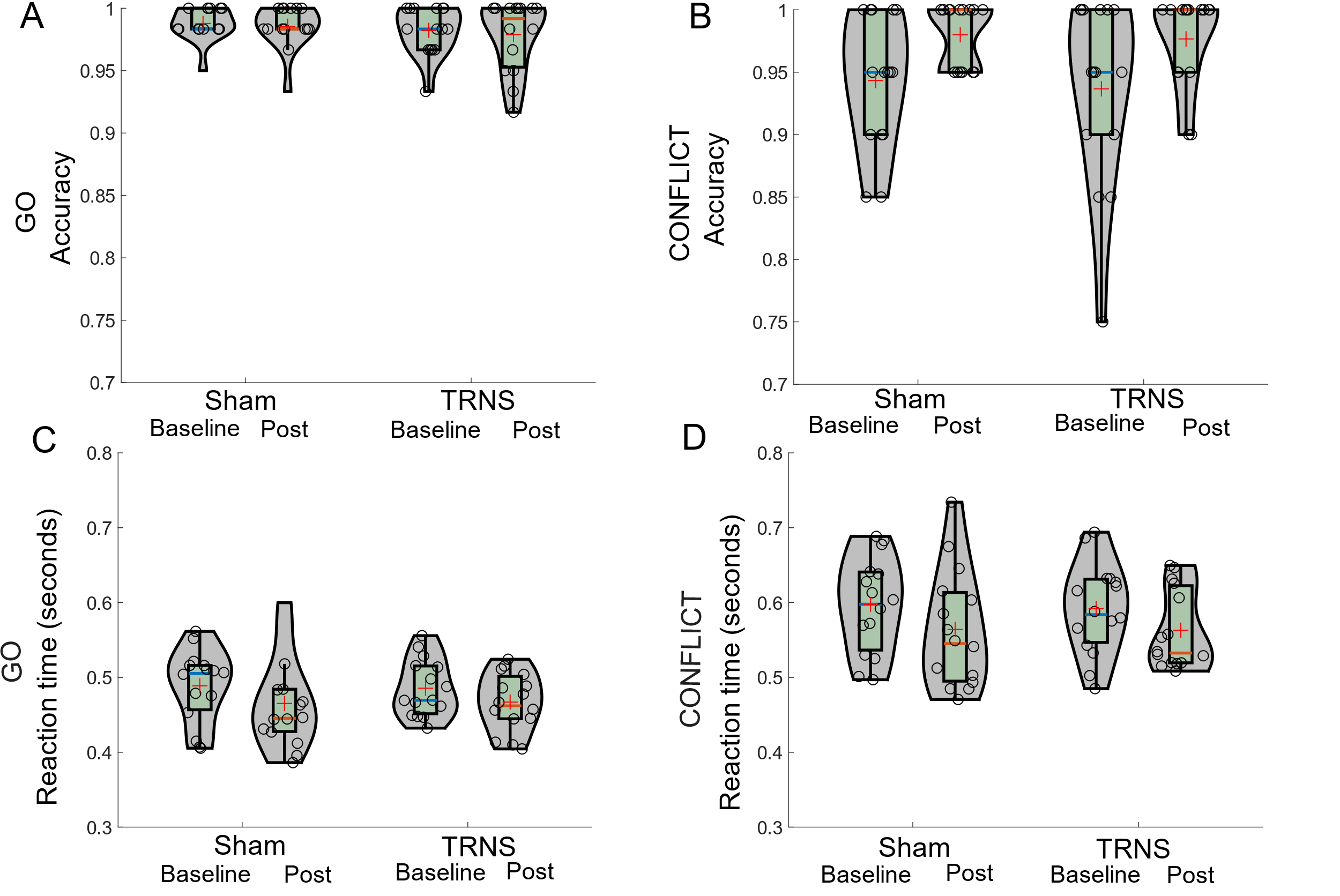
For accuracy, as hypothesized, there was no main effect of state (F(1,14) = 0.49, p = 0.49) or condition (F(1,14) = 1.39, p = 0.26) or an interaction(F(1,14) = 0.02, p = 0.88) in Go trials (Figure S3A). For Conflict trials (Figure S3B), there was a main effect of state (F(1,14) = 13.82, p = 0.002) however no main effect of condition (F(1,14) = 0.5, p = 0.7) or interaction (F(1,14) = 0.02, p = 0.89). A Bonferroni corrected post-hoc t-test for state showed an increase in accuracy after stimulation, irrespective of active or sham, compared to baseline (mean ± std error: baseline = 0.94 ± 0.01, after-stimulation = 0.98 ± 0.01, p = 0.006). This indicates the effect of practice on Conflict trials.

Figure S3 shows the accuracy and reaction time (seconds) during baseline and after-stimulation (post) for sham and transcranial random noise stimulation (TRNS) for correct Go (A &C) and correct Conflict (B & D) trials. The red crosses and blue horizontal bars on the violin plots indicate mean and median, respectively.

Similarly, for reaction time, there was a main effect of state (F(1,14) = 7.55, p = 0.02) but no main effect of condition (F(1,14) = 0.02, p = 0.92) or an interaction (F(1,14) = 0.09, p = 0.76) during correct-Go trials (Figure S3C). A Bonferroni corrected post hoc t-test for state showed a significant decrease in reaction time (p = 0.02) after stimulation (irrespective of active or sham) (0.47 ± 0.01) compared to baseline (0.49 ± 0.01). For correct Conflict trials (Figure S3D), there was a main effect of state (F(1,14) = 17.59, p<0.001) but no main effect of condition (F(1,14) = 0.08, p = 0.78) or an interaction (F(1,14) = 0.04, p = 0.85). A Bonferroni corrected post hoc t-test for state showed a significant decrease in reaction time (p<0.001) after stimulation, active or sham, (0.56 ± 0.02) compared to baseline(0.6 ± 0.01).

These results confirmed that there was no stimulation related modulation of behaviors (accuracy and reaction time) during Go and Conflict trials. Mean reaction times are summarized in Table S1 for Go and Conflict trials after excluding trials with premature incorrect responses for correct trials and participants with no errors for error trials. Table 1 also shows mean (± standard deviation) accuracy and reaction times (seconds) during Go and Conflict trials for TRNS and sham stimulation conditions for correct and error trials.

| **Parameter** | **Condition** | **Baseline**  **Go** | **After stimulation Go** | **Baseline**  **Conflict** | **After stimulation**  **Conflict** |
| --- | --- | --- | --- | --- | --- |
| Accuracy | TRNS | 0.98±0.01 | 0.98±0.03 | 0.93±0.07 | 0.98±0.04 |
|  | Sham | 0.99±0.01 | 0.99±0.02 | 0.94±0.05 | 0.98±0.02 |
| Reaction time (seconds)- Correct trials | TRNS | 0.48±0.04 | 0.47±0.04 | 0.59±0.06 | 0.56±0.05 |
|  | Sham | 0.49±0.05 | 0.47±0.06 | 0.6±0.06 | 0.56±0.08 |
| Reaction time (seconds)- Error trials | TRNS | 0.52±0.14 | 0.69±0.19 | 0.64±0.12 | 0.54±0.07 |
|  | Sham | 0.56±0.14 | 0.51±0.09 | 0.63±0.15 | 0.59±0.11 |

Table S1: Accuracy – minimum value is zero and maximum value is one indicating 0 and 100%, respectively.

*Effect of stimulation on burst characteristics*

To further understand the change in spectral power (Figure 1E) after TRNS, we extracted intermittent beta-burst features (average amplitude, average duration, and total number of bursts) at baseline and after stimulation for both TRNS and sham conditions. One participant was excluded from this analysis due to lack of a distinct beta peak. The features corresponded to average metrics per participant (Figure: S4, S5).

*Fz- Burst features*

For burst duration (milliseconds, Figure 1F), there was a main effect of state (F(1,13) = 6.36, p = 0.025) but not condition (F(1,13) = 0.16, p = 0.69) or interaction (F(1,13) = 8.91, p = 0.011). A Bonferroni corrected post hoc t-test for state showed a significant increase in burst duration (p = 0.025) after stimulation (TRNS or sham) (187.35 ± 4.82) compared to baseline (174.81 ± 4.36). A paired sample t-test also highlighted a significant increase in burst duration after TRNS (t(13) = -4.5, *p*<0.001) but not sham (t(13) = 0.32, *p* = 0.75). For burst amplitude, there was no main effect of state (F(1,13) = 3.46, p = 0.085) or condition (F(1,13) = 0.125, p = 0.73) or interaction (F(1,13) = 0.115, p = 0.74). Similarly, for the total number of bursts, there was no effect of state (F(1,13) = 0.2, p=0.66) or condition (F(1,13) = 0.15, p = 0.7) or interaction (F(1,13) = 1.17, p = 0.3).

*C_3_- Burst features*

For burst amplitude, there was no main effect of state (F(1,13) = 0.82, p = 0.38) or condition (F(1,13) = 0.001, p = 0.98) or interaction (F(1,13) = 0.03, p = 0.87). Similarly, for burst duration, there was no main effect of state (F(1,13) = 1.17, p = 0.29) or condition (F(1,13) = 0.06, p = 0.8) or interaction (F(1,13) = 0.01 p = 0.92) and for burst number, we observed no main effect of state (F(1,13) = 2.73, p = 0.12) or condition (F(1,13) = 0.58, p = 0.45) but interaction (F(1,13) = 8.27 p = 0.013). A paired sample t-test showed an increase in burst number after TRNS (t(13) = -3.56, p<0.01) but not for the sham condition (t(13) = 0.46, p = 0.65).

The plots below show the histograms of the burst characteristics (duration) for TRNS and Sham conditions at baseline and after stimulation.


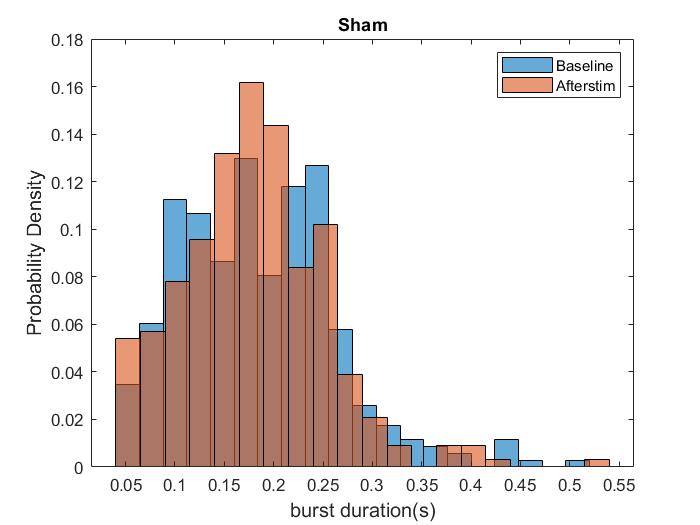

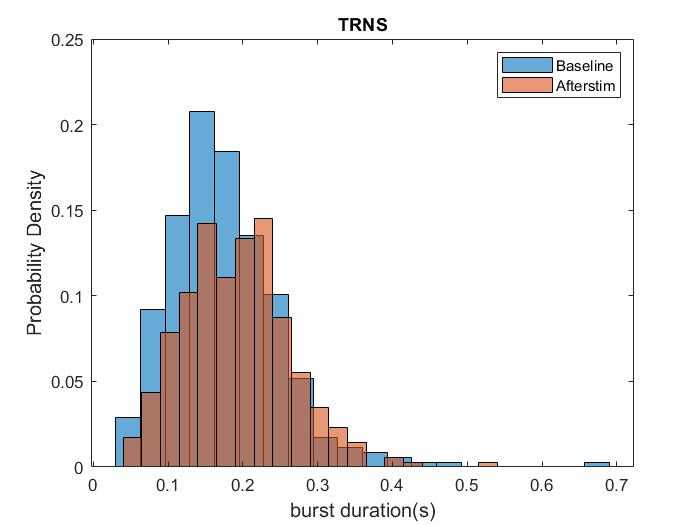

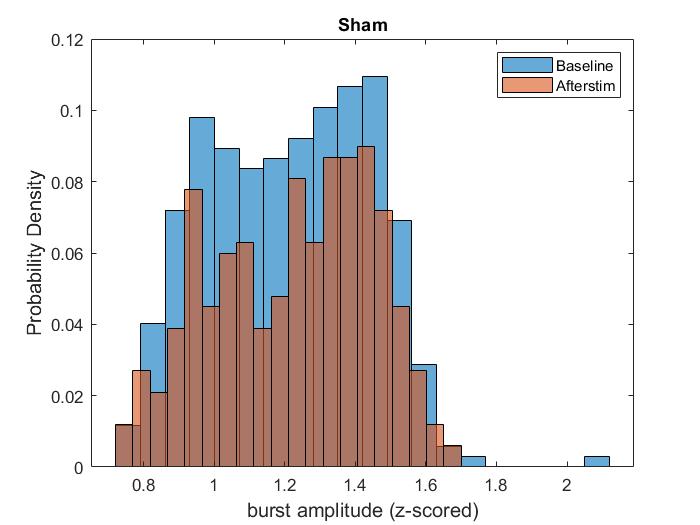

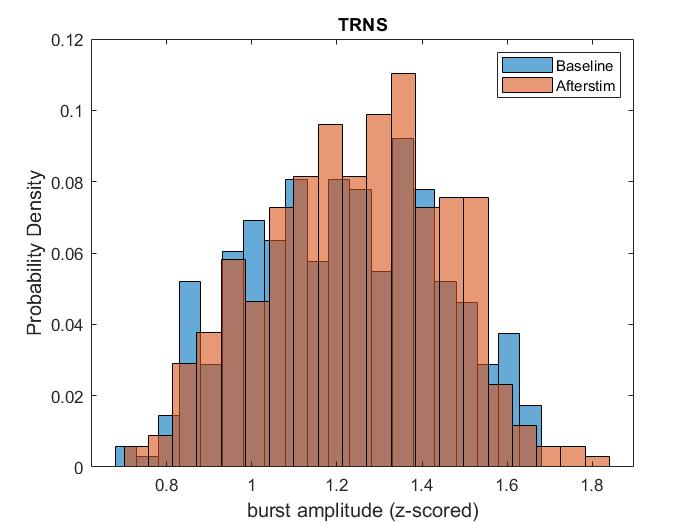


Figure S4 show the histogram of the burst duration calculated across all the participants at baseline and after stimulation for TRNS (left) and sham conditions.


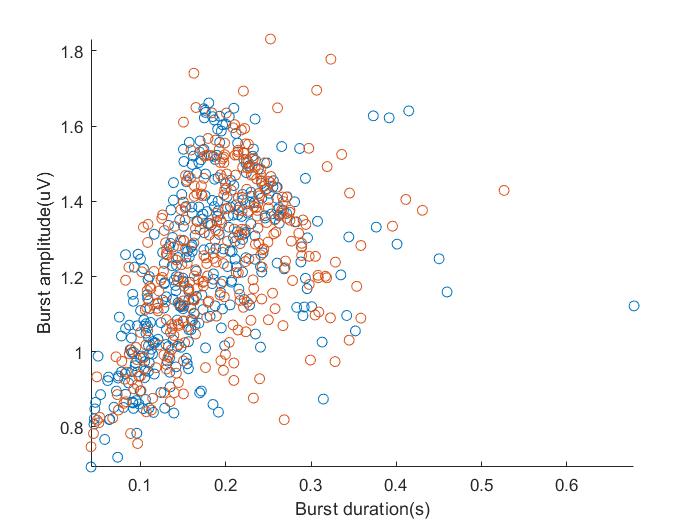

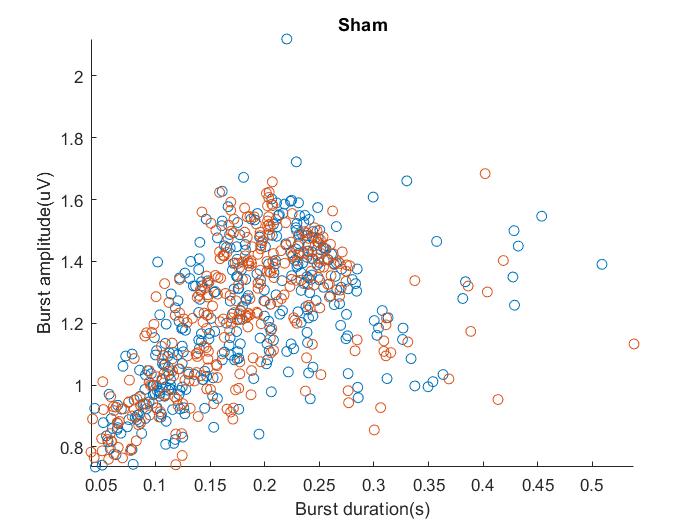


Figure S5 shows the scatter plot between burst duration and burst amplitude for TRNS (left) and sham (right) for baseline (blue) and after stimulation (red)

Table S2 summarizes beta band burst features for F_Z_ and C_3_

| **Parameter** | **Condition** | **Baseline**  **F_z_** | **After stimulation**  **F_z_** | **Baseline**  **C_3_** | **After stimulation C_3_** |
| --- | --- | --- | --- | --- | --- |
| Burst duration  (milliseconds) | TRNS | 168.54±16.61 | 196.15±29.1 | 182.02±21.58 | 177±20.81 |
|  | Sham | 181.1±27.3 | 178.55±15.18 | 183.37±24.1 | 179.25±19.25 |
| Burst amplitude (A.U) | TRNS | 1.22±0.06 | 1.25±0.08 | 1.24±0.04 | 1.25±0.06 |
|  | Sham | 1.22±0.08 | 1.24±0.07 | 1.24±0.08 | 1.1.25±0.05 |
| Number of bursts | TRNS | 23.21±3.4 | 22.86±3.3 | 22.57±2.41 | 24.71±2.67 |
|  | Sham | 23.1±2.58 | 23.86±2.65 | 23.07±2.58 | 22. 71±3.05 |
| Burst Threshold | TRNS | 0.76±0.04 | 0.74±0.03 | 0.72±0.05 | 0.74±0.05 |
|  | Sham | 0.74±0.05 | 0.75±0.05 | 0.74±0.05 | 0.76±0.04 |

*Effect of stimulation on ERP*

’*ITI-corrected epoch’* data was averaged across trials per participant to obtain an average ERP which showed significant differences in the P3 potential (Figure S6). Non-parametric cluster-based statistics identified significant clusters (*p* < 0.005 after Bonferroni corrections across 6 conditions) over F_Z_ between the Go and No-Go (Figure S6A and S6B) conditions during sham, with a higher amplitude P3 for the No-Go condition. Although we observed an effect of TRNS between No-Go and conflict (p = 0.01) trials, this did not survive multiple comparisons. There was also a significant increase in P3 between Go and No-Go, and Conflict and No-Go conditions after TRNS but not sham (Figure S6C and S6D) over the motor cortex (C_3_). It should be noted that there were no significant differences in the N2 potential.


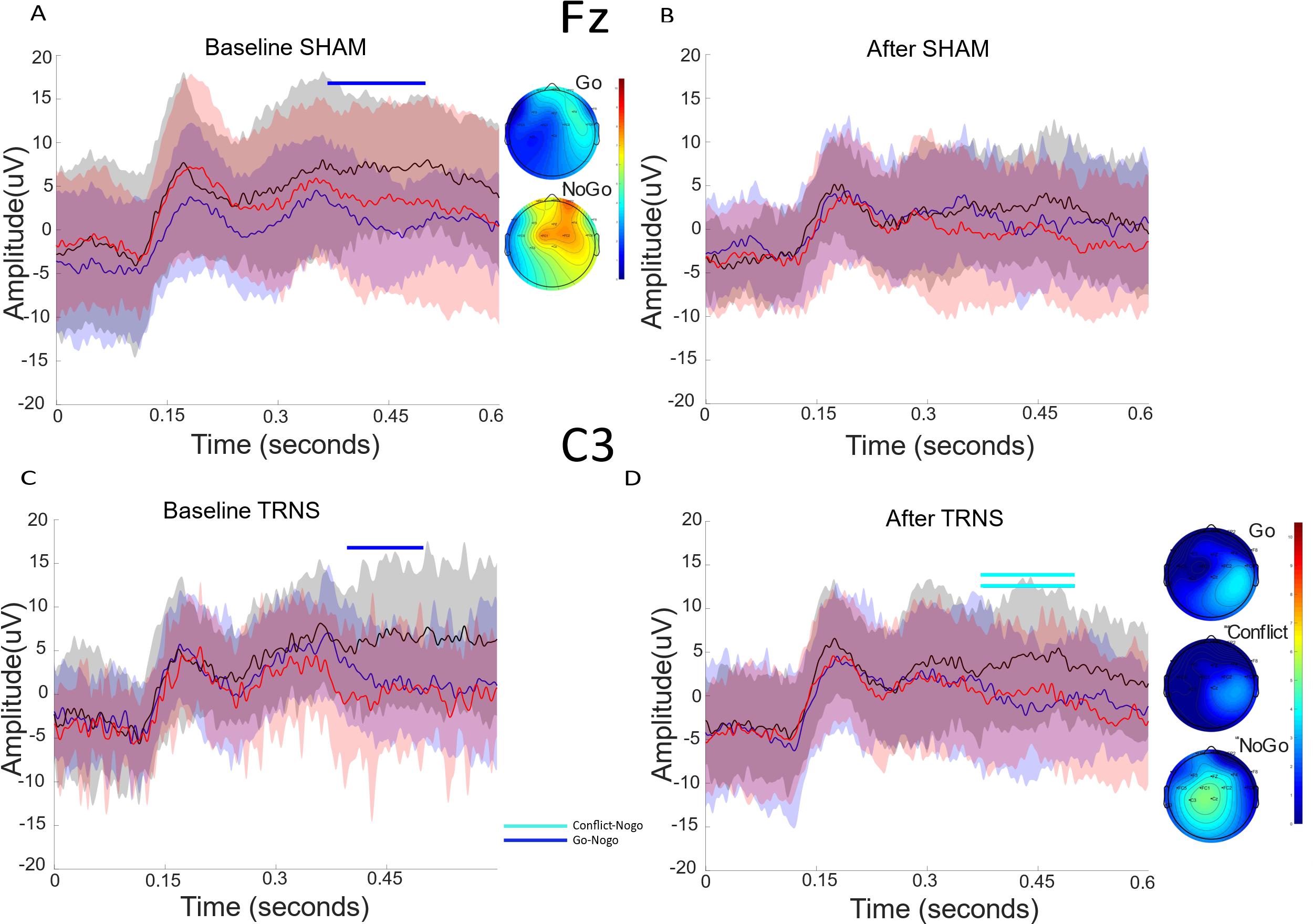


Figure S6 shows the event related potentials (ERP) across the three conditions (blue- Go, red-conflict and black-No-Go) at baseline and after-stimulation for Fz (A & B) and C3 (C & D) respectively. The x-axis corresponds to time in seconds and y-axis indicates the amplitude (µV). The bar line indicates the duration when a significant difference has been observed after correcting for multiple comparisons (Blue -Go and No-Go, Cyan-Conflict and No-Go). The topo plots in panels A & D display the average activity between 300-500milliseconds. The topo plot in panel A compares the activity between Go and No-Go condition at baseline and panel D shows Go, Conflict and No-Go (from top to bottom) after TRNS stimulation measured across C3.

For several decades, P3 potentials (occurring between 300 and 800ms after cue presentation) have been associated with rare events such as No-Go trials and known to capture inhibitory control. We observed a differential effect of TRNS on P3 across the three conditions: P3 amplitude over the motor cortex was significantly higher for No-Go compared to Go and Conflict trials after TRNS, suggesting a higher inhibitory control driving the improvement in No-Go accuracy. We did not observe any differences at the level of N2 between Go, Conflict or No-Go trials, suggesting a conflict monitoring response to all cues.

**References**

[1] Cyders MA, Littlefield AK, Coffey S, Karyadi KAJAb. Examination of a short English version of the UPPS-P Impulsive Behavior Scale. 2014;39(9):1372-6.

[2] Fertonani A, Ferrari C, Miniussi CJCN. What do you feel if I apply transcranial electric stimulation? Safety, sensations and secondary induced effects. 2015;126(11):2181-8.

[3] Zavala B, Jang A, Trotta M, Lungu CI, Brown P, Zaghloul KA. Cognitive control involves theta power within trials and beta power across trials in the prefrontal-subthalamic network. Brain 2018;141(12):3361-76.

[4] Szul MJ, Bompas A, Sumner P, Zhang J. The validity and consistency of continuous joystick response in perceptual decision-making. Behavior Research Methods 2020;52:681-93.

[5] Delorme A, Makeig SJJonm. EEGLAB: an open source toolbox for analysis of single-trial EEG dynamics including independent component analysis. 2004;134(1):9-21.

[6] de Cheveigné A. ZapLine: A simple and effective method to remove power line artifacts. NeuroImage 2020;207:116356.

[7] Bell AJ, Sejnowski TJ. An information-maximization approach to blind separation and blind deconvolution. Neural computation 1995;7(6):1129-59.

[8] Delorme A, Makeig S. EEGLAB: an open source toolbox for analysis of single-trial EEG dynamics including independent component analysis. Journal of neuroscience methods 2004;134(1):9-21.

[9] Lopez-Calderon J, Luck SJJFihn. ERPLAB: an open-source toolbox for the analysis of event-related potentials. 2014;8:213.

[10] Oostenveld R, Fries P, Maris E, Schoffelen J-MJCi, neuroscience. FieldTrip: open source software for advanced analysis of MEG, EEG, and invasive electrophysiological data. 2011;2011.

[11] Cagnan H, Mallet N, Moll CK, Gulberti A, Holt AB, Westphal M, et al. Temporal evolution of beta bursts in the parkinsonian cortical and basal ganglia network. Proceedings of the National Academy of Sciences 2019;116(32):16095-104.

[12] Tinkhauser G, Pogosyan A, Tan H, Herz DM, Kühn AA, Brown P. Beta burst dynamics in Parkinson’s disease OFF and ON dopaminergic medication. Brain 2017;140(11):2968-81.

[13] Donoghue T, Haller M, Peterson EJ, Varma P, Sebastian P, Gao R, et al. Parameterizing neural power spectra into periodic and aperiodic components. Nature neuroscience 2020;23(12):1655-65.

[14] West TO, Duchet B, Farmer SF, Friston KJ, Cagnan H. When do bursts matter in the primary motor cortex? Investigating changes in the intermittencies of beta rhythms associated with movement states. Progress in Neurobiology 2023;221:102397.

[15] Oostenveld R, Fries P, Maris E, Schoffelen J-M. FieldTrip: open source software for advanced analysis of MEG, EEG, and invasive electrophysiological data. Computational intelligence and neuroscience 2011;2011.

[16] Zavala B, Jang A, Trotta M, Lungu CI, Brown P, Zaghloul KAJB. Cognitive control involves theta power within trials and beta power across trials in the prefrontal-subthalamic network. 2018;141(12):3361-76.
